# Optical Coherence Tomography in Modern Ophthalmology: Innovations Across the Anterior and Posterior Segment

**DOI:** 10.3390/diagnostics16081114

**Published:** 2026-04-08

**Authors:** Ninel Z. Gregori

**Affiliations:** Bascom Palmer Eye Institute, Department of Ophthalmology, University of Miami Miller School of Medicine, Miami, FL 33136, USA; ngregori@med.miami.edu; Tel.: +1-305-326-6021

The invention of optical coherence tomography (OCT) in the late 1980s marked a turning point in ophthalmology, fundamentally transforming how retinal diseases are diagnosed, monitored, and treated. By enabling noninvasive, micrometer-resolution, cross-sectional living biopsy imaging, the technology enabled us to visualize microstructural changes such as macular edema, retinal thinning, and vitreoretinal interface abnormalities. This unprecedented level of detail not only improved our ability to detect diseases early and diagnose more accurately but also provided objective, quantitative biomarkers that could be tracked over time to monitor diseases and fine-tune treatment protocols. Consequently, the OCT became central to the development and validation of new therapies, allowing researchers to assess treatment response and refine surgical techniques.

Optical coherence tomography (OCT) is a noninvasive imaging technology that uses low-coherence interferometry to produce a two-dimensional image of optical scattering from internal tissue microstructures in order to generate high-resolution, cross-sectional images in transparent and turbid media. Its development began in the 1980s through pioneering work in low-coherence interferometry by researchers including James Fujimoto, alongside key contributions from David Huang, Joel Schuman, Carmen Puliafito, and others at Massachusetts Institute of Technology (MIT), Massachusetts Eye and Ear Infirmary, and Massachusetts General Hospital [1,2]. The first live retinal images were published in 1993 [3]. Early time-domain OCT systems, introduced clinically in 1996 by Zeiss after acquiring the technology from MIT, provided moderate resolution and were limited by slow acquisition speeds. The advent of spectral-domain OCT in the mid-2000s significantly improved imaging speed and resolution, while more advanced swept-source OCT systems later enabled deeper tissue penetration and faster scanning. More recently, OCT angiography expanded OCT’s capabilities by allowing noninvasive visualization of retinal and choroidal vasculature. Over just two decades, OCT has become a cornerstone of modern ophthalmic imaging in posterior and anterior segments of the eye, offering high-resolution, cross-sectional visualization of ocular microstructures. When integrated with modalities such as fundus photography, fundus autofluorescence, and fluorescein angiography, OCT provides structural details, enabling clinicians to detect subtle pathological changes, monitor disease progression, and tailor treatments with greater precision. Recent advances in OCT technology have transformed clinical practice and research alike, enhancing diagnostic precision, surgical planning, and postoperative monitoring while contributing to a more individualized treatment approach and better visual outcomes for our patients.

This Special Issue highlights a range of studies showcasing the expanding role of OCT in both anterior and posterior segment imaging. The articles include original research and reviews utilizing advanced modalities such as spectral-domain and swept-source OCT and OCT angiography to investigate retinal diseases and corneal pathology. Topics covered include OCT-based analysis of macular hole closure mechanisms, identification of OCT-derived biomarkers for retinal detachment to guide surgical planning and prognosis, and assessment of neuroretinal changes following COVID-19 disease. Additional contributions explore emerging applications such as intraoperative and home-based OCT, preoperative screening of corneal grafts with standardized grading of interface abnormalities to improve graft preparation and maximize visual outcomes, as well as optimization of anterior segment imaging techniques in drug reservoir implants. Collectively, these studies demonstrate the versatility of OCT in improving diagnostic precision, enhancing medical and surgical decision-making, and advancing understanding of ocular disease, as well as highlighting areas of further research.

While these publications point out that OCT remains an indispensable tool in the management of various ocular conditions and surgery, further research in the application of OCT to the management of retinal and anterior segment conditions must address several critical gaps. Currently, axial and lateral resolution constraints limit the ability to distinguish fine microstructural changes and cellular details [4]. Shadowing artifacts, signal attenuation, and limited penetration in highly pigmented or edematous tissues further restrict image quality. While newer technologies such as adaptive optics-enhanced OCT and ultrahigh-resolution systems show promise, their clinical adoption is not yet widespread, leaving a gap between research capabilities and everyday clinical practice. Further research is needed to establish standardized imaging protocols, methodology, and nomenclature, which are essential for the ability to reproduce and compare results across studies. Large multicenter trials are needed to validate various OCT-derived biomarkers and to develop prognostication and best treatment practices. More recent advances in high-resolution imaging techniques, including adaptive optics OCT and en face OCT, stand to improve our ability to visualize retinal microarchitecture and correlate biomarkers with functional outcomes and should be studied further. Integration of machine learning and artificial intelligence into OCT image analysis should be developed to improve our ability to objectively detect subtle anatomical features, compare them across various follow-up time points, and develop more precise treatment techniques with the overarching goal of advancing our understanding of disease pathophysiology and optimizing patient outcomes.
